# Orientisargidae fam. n., a new Jurassic family of Archisargoidea (Diptera, Brachycera), with review of Archisargidae from China

**DOI:** 10.3897/zookeys.238.3624

**Published:** 2012-11-06

**Authors:** Junfeng Zhang

**Affiliations:** 1 College of Palaeontology, Shenyang Normal University, Shenyang, 110034, P. R.China; 2 Nanjing Institute of Geology and Palaeontology, Chinese Academy of Sciences, Nanjing 210008, P. R. China

**Keywords:** Diptera, Orientisargidae fam. n., *Orientisargus illecebrosus* gen. et sp. n., Archisargidae, *Daohugosargus* gen. n., Uranorhaginidae, Jurassic and Early Cretaceous, China

## Abstract

A pair of fly impressions is described as a new species of a new genus, *Orientisargus illecebrosus* **gen. et sp. n.**, referred to a new family Orientisargidae **fam. n.** within Archisargoidea of Brachycera, Diptera. The systematic position of Orientisargidae is discussed. *Daohugosargus* **gen. n.** is proposed for *Sharasargus eximius* KY Zhang et al., 2008. Uranorhagionidae is a junior synonym for Archisargidae. Meanwhile, Mostovskisarginae is a junior synonym for Uranorhagionidae. *Mostovskisargus* JF Zhang, 2010 and *Strenorhagio* KY Zhang et al., 2010 are synonymized with *Uranorhagio* KY Zhang et al., 2010. *Uranorhagio* includes three species: *Uranorhagio asymmetricus* (KY Zhang et al., 2010), **comb. n.**, *Uranorhagio daohugouensis* KY Zhang et al., 2010 and *Uranorhagio deviatus* (KY Zhang et al., 2010), **comb. n.** *Strenorhagio grimaldi* KY Zhang et al., 2010 is synonymous with *Uranorhagio deviatus*. *Mostovskisargus portentosus* JF Zhang, 2010, *Mostovskisargus signatus* JF Zhang, 2010 and *Strenorhagio conjugovenius* KY Zhang et al., 2010 are synonymous with *Uranorhagio asymmetricus*. *Brevisolva* KY Zhang et al., 2010 is a junior synonym for *Mesosolva* Hong, 1983. A new specific name, *Mesosolva zhangae* **nom. n.**, is proposed for *Brevisolva daohugouensis* KY Zhang et al., 2010. *Mesosolva jurassica* KY Zhang et al., 2010 should be synonymized under *Mostovskisargus sinensis* KY Zhang et al., 2010. *Sinallomyia* nom. n. is proposed for *Allomyia* Ren, 1998. The systematic positions for *Helempis eucalla* Ren, 1998, *Helempis yixianensis* Ren, 1998, *Pauromyia oresbia* Ren, 1998 and *Sinallomyia ruderalis* (Ren, 1998) are reassessed. These taxa belong to Archisargidae rather than to Tabanidae, Rhagionidae and Protempididae, respectively.

## Introduction

The superfamily Archisargoidea comprises three families: Archisargidae, Kovalevisargidae and Eremochaetidae. The Archocyrtidae is probably the fourth family of archisargoids (Mostovski 1997). It is interesting that Archisargidae and Kovalevisargidae constitute one sister group; whereas Eremochaetidae and Archocyrtidae probably form another sister group. All these families synchronously appeared in the Callovian–Oxfordian from the Karabastau Formation at the Karatau-Mikhailovka locality in the Karatau Mountain Ridge, Chimkent Region, South Kazakhstan Province, Kazakhstan. To date, however, only the representatives of archisargids and eremochaetids are known to extend into the Early Cretaceous.

Lately, thousands of brachycerans have been discovered from the “Daohugou Formation” in the vicinity of Daohugou, Ningcheng, Chifeng, Inner Mongolia (JF Zhang and HC Zhang 2003; JF Zhang 2010a, 2010b, 2010c, 2011a, 2011b, 2012a, 2012b, in press; JF Zhang and Li 2012; KY Zhang et al. 2006, 2007a, 2007b, 2008a, 2008b, 2008c, 2008d, 2009, 2010a, 2010b) and the Yixian Formation in the vicinity of Huangbanjigou, Shangyuan, Beipiao, Liaoning, China (Ren et al. 1995; Ren 1998; Huang and Lin 2006). It is interesting that the members of almost all the archisargid and kovalevisargid genera recorded from the Karabastau Formation were also recovered from the “Daohugou Formation”. Meanwhile, on the basis of review of brachycerans from the Yixian Formation, it is clear that some relics of archisargid genera and species did also occur in the Early Cretaceous (see Discussion below).

A new family, Orientisargidae fam. n., composed of a new genus and species, *Orientisargus illecebrosus* gen et sp. n., is described here. Another new genus, *Daohugosargus* gen. n., is proposed for the known species *Sharasargus eximius* KY Zhang et al., 2008. *Daohugosargus eximius* (KY Zhang et al., 2008), comb. n. has a characteristic wing venation, which differs sharply from all the known representatives of archisargids, and may be temporarily assigned to the subfamily Uranorhagioninae (stat. n.) within Archisargidae. The systematic position for Uranorhagionidae is reassessed. It is a junior synonym for Archisargidae, and may be degraded as a subfamily within Archisargidae. Mostovskisarginae is a junior synonym for Uranorhagionidae. Meanwhile, *Mostovskisargus* JF Zhang, 2010 and *Strenorhagio* KY Zhang et al., 2010 can be synonymized with *Uranorhagio* KY Zhang et al., 2010. Two species, *Strenorhagio deviatus* KY Zhang et al., 2010 and *Strenorhagio grimaldi* KY Zhang et al., 2010, can be united into one species: *Uranorhagio deviatus* (KY Zhang et al., 2010), comb. n. *Mostovskisargus portentosus* JF Zhang, 2010, *Mostovskisargus signatus* JF Zhang, 2010 and *Strenorhagio conjugovenius* KY Zhang et al., 2010 are synonyms for *Uranorhagio asymmetricus* (KY Zhang et al., 2010), comb. n. The species of *Mesosolva* and related taxa recently described from the “Daohugou Formation” are reassessed: *Brevisolva* KY Zhang et al., 2010 is a junior synonym for *Mesosolva* Hong, 1983. A new specific name, *Mesosolva zhangae* nom. n., is proposed for the *Brevisolva daohugouensis* KY Zhang et al., 2010. *Mesosolva jurassica* KY Zhang et al., 2010 should be synonymized under *Mesosolva sinensis* KY Zhang et al., 2010. A new generic name, *Sinallomyia* nom. n., is proposed instead of *Allomyia* Ren, 1998 which is a junior homonym for *Allomyia* Banks, 1916 (a genus of Trichoptera). The Early Cretaceous *Helempis eucalla* Ren, 1998, *Helempis yixianensis* Ren, 1998, *Pauromyia oresbia* Ren, 1998 and *Sinallomyia ruderalis* (Ren, 1998) from the Yixian Formation previously placed, respectively, in the Tabanidae, Rhagionidae and Protempididae should be transferred to the Archisarginae of Archisargidae.

## Material and methods

Specimen descriptions, photographs, and drawings were obtained without the application of glycerol to the surface of the specimens. The specimens collected by the author in field were examined under a stereomicroscope (Wild Heerbrugg) and illustrated with the aid of a drawing tube attached to it, re-adjusted using image-editing software (Adobe Photoshop CS). The digital photographs were taken using stereomicroscope (AXioCamHR3).

Wing venation terminology here follows Wootton and Ennos (1989), and Shcherbakov et al. (1995). The cell traditionally named the anal cell is, in fact, considered to be the cubital cell herein. The specimens are deposited in the Nanjing Institute of Geology and Palaeontology (NIGP), Chinese Academy of Sciences.

## Systematics

### Superfamily Archisargoidea Rohdendorf, 1962

#### Family Orientisargidae fam. n.

urn:lsid:zoobank.org:act:6E5DC307-FFD3-48A6-895E-12047C8A9001

http://species-id.net/wiki/Orientisargidae

##### Type genus.

*Orientisargus* gen. n.

##### Included genus.

The type genus only.

##### Diagnosis.

Large sized (more than 12 mm) flies. Legs and abdomen slender, strongly pubescent but devoid of bristles; antennal pedicel longest, first flagellomere neither segmented nor arista or style on its tip; wing narrow and long, subpetiolate, alula absent; venation not costalized; R pectinate; C running around entire wing margin although thinned beyond wing tip; C, Sc, R and CuA strong; Sc, R1 and R2+3 long; R4+5 simple (not bifurcated); origin of Rs proximal, nearly at level of M fork; discoidal cell slightly shifted distally; crossvein m-cu absent; M3+4 stem strongly flexed, and touching CuA; cell m3 closed; hind legs stout and long, tibial spurs well developed, empodium wanting; female cerci foliaceous.

##### Remarks.

This new family demonstrates similar body structures and wing venation to the family Archisargidae Rohdendorf, 1962 based on the following characters: large sized flies with hind legs and abdomen stout and long, body strongly pubescent but devoid of bristles; wing narrow and long, subpetiolate, alula absent; venation: C running around wing margin although thinned beyond wing tip; long and strong Sc and R1 (R1 more than four-fifths of wing length, and clearly stouter than M); and the position of r-m, which meets R4+5 and fore margin of d, respectively; as well as the position of disciodal cell, which is more or less shifted distally. However, from all known representatives of Archisargidae it differs by the simple R4+5, which is not bifurcated, the origin of Rs which is clearly proximal, the strongly flexed M3+4 stem, of which bM3+4 section becomes short, crossvein-like, and touches CuA instead of m-cu; Furthermore, considering the characteristic features that R4+5 is simple, R furcated pectinately and the origin of Rs is proximal, Orientisargidae fam. n. is similar to the family Kovalevisargidae Mostovski, 1997, the sister group of Archisargidae within Archisargoidea. It is distinct from all the kovalevisargids in having longer Sc, R1 and R2+3, the closed cell m3, and the absence of m-cu. It is interesting that the long pedicel, the absence of arista or style on the tip of antenna, the absence of empodium may be the unique features of Orientisargidae and are found neither in Archisargidae nor in Kovalevisargidae. It is also interesting that the new family has a pair of foliaceous cerci on the female terminalia, which is only present in Uranorhagioninae (=Mostovskisarginae) within Archisargidae (JF Zhang, 2010a).

**Figure 1.**
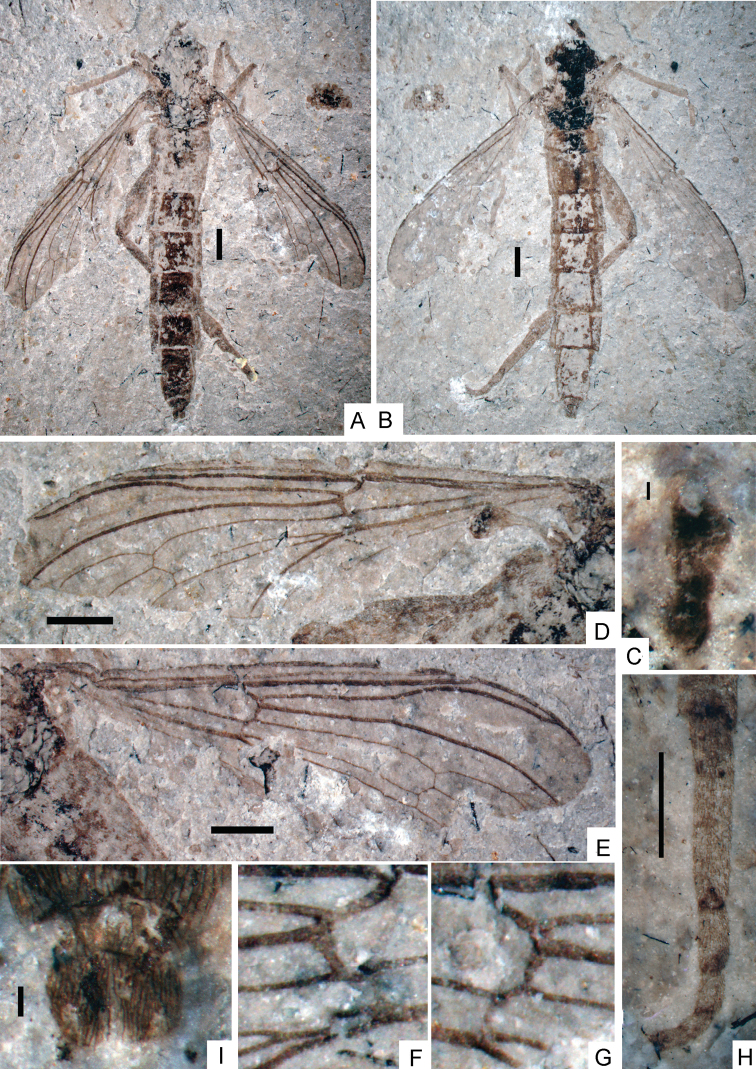
*Orientisargus illecebrosus* gen et sp. n., NIGP DHG901a, NIGP DHG901b, part and counterpart, holotype, photographs, female, dorsal view. **A B** body **C** antenna **D** left wing **E** right wing **F** part enlarged of left wing **G** part enlarged of right wing **H** tarsus of hind leg **I** abdominal apex of female. Scale bars represent 1 mm except for C and I for which scale bars represent 0.1 mm.

As for m-cu is concerned, an alternative explanation is possible that a very short, but thick, rudimentary m-cu connecting CuA and flex point of M3+4 is present (see Figure 1F, G). In such case, however, it becomes too short to measure.

#### Genus Orientisargus gen. n.

urn:lsid:zoobank.org:act:670D9E14-08A2-4352-9297-28137CE511F7

http://species-id.net/wiki/Orientisargus

##### Type species.

*Orientisargus illecebrosus* sp. n.

##### Included species.

The type species only.

##### Derivation of name.

Latin, *orient-*, oriental, alluding to the origin of the fossils, and *sargus*, a common ending in archisargid genera (the masculine gender).

##### Diagnosis.

First antennal flagellomere conical. R2+3 arched medially, ending in C before wing tip, and far from R1 end. R4+5 ending beyond wing tip. Rs stem and bR4+5 short. Origin of Rs nearly at level of d base. Rs fork shifted distally of M fork. Crossvein r-m meeting R4+5 and M1+2, near to d base. Four medial veins present. Cells d and m3 narrow and long, the latter cell with long petiole. Section bM3+4 shorter than r-m. CuA and CuP subparallel, and cu cell (traditionally anal cell) wide open.

##### Remarks.

Usually, the generic diagnosis is covered by the familial diagnosis when the family comprises only one genus. Nevertheless, the familial diagnosis can be well defined based on the characteristics of its sister groups Archisargidae and Kovalevisargidae. In such case, a generic diagnosis is temporarily proposed but need to be revised when another new genus (or genera) within the new family has (have) been discovered.

#### Orientisargus illecebrosus sp. n.

urn:lsid:zoobank.org:act:08E30A96-3B6C-4705-B7BC-26CBF0A791A8

http://species-id.net/wiki/Orientisargus_illecebrosus

Figures 1 [Fig F2] –3

##### Derivation of name.

Latin, *illecebrosus*, enchanting, alluding to the special wing venation.

**Holotype:** NIGP DHG901a, NIGP DHG901b, part and counterpart, a pair of nearly complete female archisargoid flies, is held in the Nanjing Institute of Geology and Palaeontology, Chinese Academy of Sciences.

##### Type area and horizon.

“Daohugou Formation”, in the vicinity of Daohugou, Ningcheng, Inner Mongolia, China (uppermost Middle Jurassic – lowermost Upper  Jurassic).

##### Repository.

The Nanjing Institute of Geology and Palaeontology, Chinese Academy of Sciences.

##### Description.

Female insect relatively slender and long. Head and thorax dark brown, otherwise yellowish brown. Head large, semiglobose. Antenna stout, scape ovate, pedicel trapeziform, apically wider than basally, flagellomere short and stout with rounded tip, slightly wider than long. Thorax subovate, clearly longer and wider than head. Wing 3.8 times longer than wide, all veins are markedly thickened except M and CuP, which become moderately thin. Sc nearly four-fifths wing length. R1 straight, more than four-fifths wing length. Origin of Rs slightly basal to midpoint of wing or M fork. Rs stem slightly longer than r-m, and less than one-tenth R2+3 length. Basal section of R2+3 straight, distal section clearly arched forward, ending near to wing tip. Rs fork a little distad to M fork. Section bR4+5 very short, dR4+5 arched, and nearly as long as R2+3. Crossvein r-m dividing anterior margin of discoidal cell as 1:21. Discoidal cell about one-third wing length, and 11 times longer than wide. M1 slightly arched forward, and more than one half diacoidal cell length. M2 straight. Section bM2 some as long as m-m. Section bM3+4 as long as bM1+2, and about one half dM3+4 length. Cell m3 rather narrow and long, some 10 times longer than wide. Section dM3 short, about one half m-m length, and nearly perpendicular to M4. Petiole of cell m3 straight, some one-third cell m3 length. Halter relatively short and stout, club subovate, some one-third halter length. Abdomen cylindrical, more than three times longer than head and thorax combined. Hind leg stout and long. Femur clavate, a little shorter, but stouter, than tibia. Tibial spurs slender and long, nearly one-third basitarsus length. Ratio of tarsomeres 1:0.43:0.28:0.11:0.17, basitarsus nearly as long as remainder tarsomeres combined. Claws small, slender. Female cerci small, longer than ninth tergite.

**Figure 2. F2:**
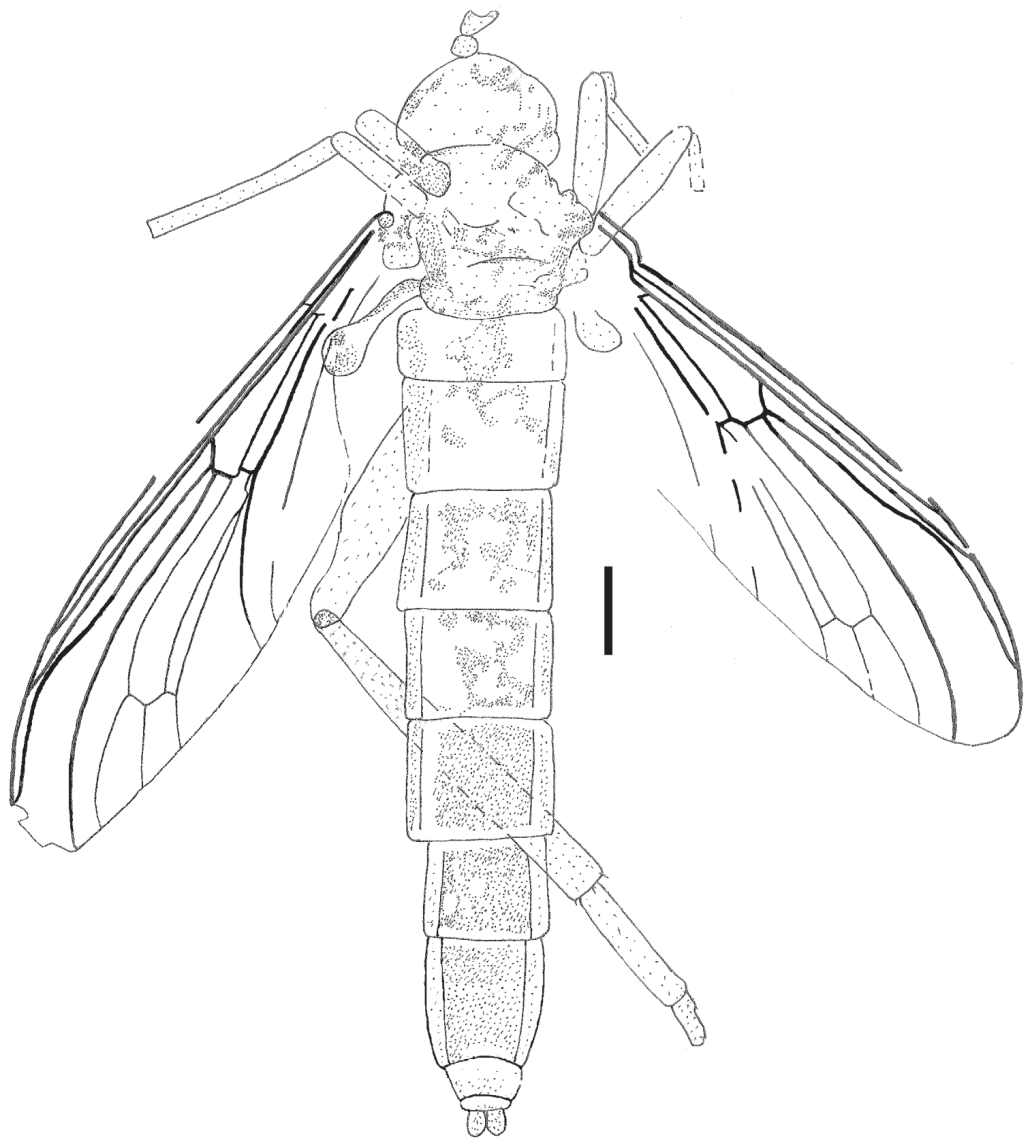
*Orientisargus illecebrosus* gen et sp. n., body, line drawing of holotype NIGP DHG901a. Scale bars represent 1 mm.

**Figure 3.**
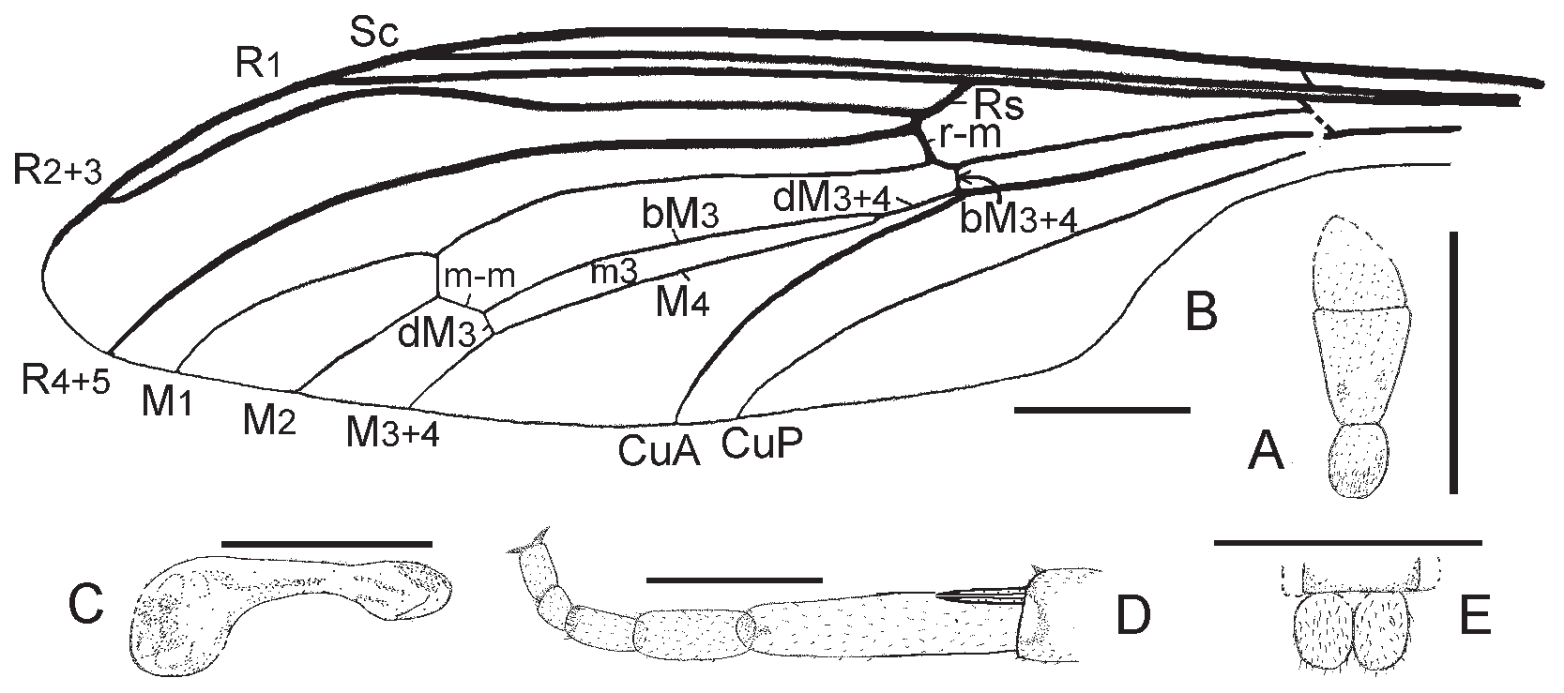
*Orientisargus illecebrosus* gen et sp. n., line drawings, **A** antenna of holotype NIGP DHG901b **B** left wing of holotype NIGP DHG901a (vein sections bM1+2, bM3+4 and dM3+4 reconverted based on right wing) **C** halter of holotype of NIGP DHG901a **D** tarsus of hind leg of holotype NIGP DHG901b **E** female cerci of holotype NIGP DHG901a. Scale bars represent 1 mm.

##### Dimensions. 

Length of body 12.2 mm; head, 1.0 mm; thorax, 2.0 mm; abdomen, 9.2 mm. Length of wing, 8.4 mm; width of wing, 2.2 mm. Length of femur of hind leg, c. 4.0 mm; tibia, 4.4 mm; tarsus, 3.2 mm.

#### Family Archisargidae

Rohdendorf, 1962

http://species-id.net/wiki/Archisargidae

Uranorhagionidae KY Zhang, Yang et Ren, 2010, p. 564, syn. n.
Origoasilidae KY Zhang, Yang et Ren, 2011, p. 995, syn. n.

##### Type genus.

*Archisargus* Rohdendorf, 1938

##### Included subfamilies.

Archisarginae Rohdendorf, 1962 and Uranorhagioninae KY Zhang, Yang et Ren, 2010, stat. n. (=Mostovskisarginae JF Zhang, 2010, syn. n.).

##### Redefinition.

**Figure 4.**
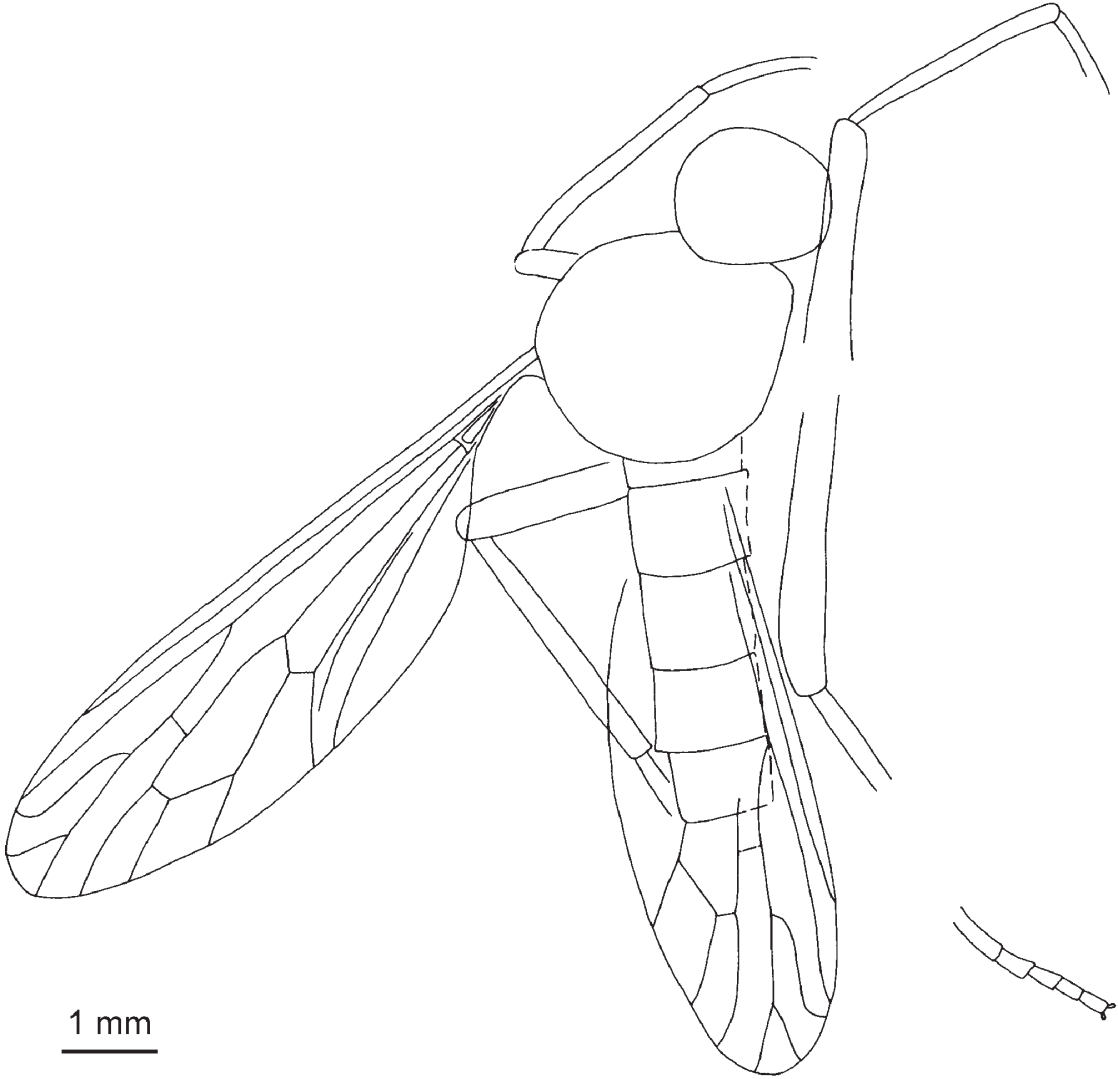
*Daohugosargus eximius* (KY Zhang, Yang et Ren, 2008) comb. n. (originally *Sharasargus eximius* KY Zhang, Yang et Ren, 2008; after KY Zhang et al. 2008).

Moderate- to large-sized flies. Body robust but usually narrow and long, strongly pubescent but devoid of bristles; first flagellomere of antenna unsegmented, arista well developed at tip of first flagellomere; hind legs stout and long, femora clavate; tibial spurs and pulvilliform empodium well developed; wing narrow and long, subpetiolate, alula absent; all longitudinal veins well developed, ending at wing margin; C running around entire wing margin although thinned near to, or beyond, wing tip, C and R strong, Sc and R1 long, R4+5 bifurcated, R2+3 usually straight and long but in some specific members R2+3 short and significantly curved [see Figure 4, *Daohugosargus eximius* (KY Zhang et al., 2008) comb. n., originally *Sharasargus eximius* KY Zhang et al., 2008], in most representatives crossvein r-m meeting R4+5 distad to Rs fork but in some specific members far basad to Rs fork (see Figure 4, *Daohugosargus eximius*), origin of Rs usually basad to, but in some specific members distad to, d base (see Figure 4, *Daohugosargus eximius*), discoidal cell shifted distally of wing midpoint.

##### Remarks.

Rohdendorf (1962) defined the family Archisargidae based on a single poorly preserved wing from the Callovian–Oxfordian Karabastau Formation. Kovalev (1981) argued that this family was described from very poor material and thus nothing definite can be said about its systematic position. However, the type genus *Archisargus* Rohdendorf, 1938, the only representative of the Archisargidae, clearly has little in common with the Jurassic Rhagionidae: it is a large fly with a long (16 mm) narrow wing. Mostovski (1996a, 1996b) described many new species referred, respectively, to some new genera or the known genera and assigned these to Archisargidae. Immediately after these, he re-described the type species of type genus based on the holotype; and a redefinition of the Archisargidae was proposed: wing venation not, or slightly, costalized; C running around entire wing margin although thinned beyond wing tip or R4 end; R1 long; R4+5 bifurcated; crossvein r-m meeting R4+5 distad to Rs fork; base of discoidal cell distad to origin of Rs from R; M4, if present, connecting with discoidal cell (Mostovski 1997).

Recently, numerous well preserved archisargid flies have been recovered from the Daohugou biota, China (JF Zhang and HC Zhang 2003; JF Zhang 2010a, 2012b; KY Zhang et al. 2007a, 2007b, 2008a, 2009, 2010a, 2010b). The familial diagnosis may be further supplemented based on information derived from these new results.

#### Subfamily Archisarginae

Rohdendorf, 1962

##### Type genus:

*Archisargus* Rohdendorf, 1938

##### Included genera.

*Archirhagio* Rohdendorf, 1938, *Archisargus* Rohdendorf, 1938, *Calosargus* Mostovski, 1997, *Flagellisargus* JF Zhang, 2012, *Mesosolva* Hong, 1983 (=*Prosolva* Hong, 1983; *Brevisolva* KY Zhang et al., 2010, syn. n.), *Origoasilus* KY Zhang et al., 2011, *Ovisargus* Mostovski, 1996 (=*Helempis* Ren, 1998, syn. n.), *Parvisargus* Mostovski, 1996, *Sharasargus* Mostovski, 1996 (=*Pauromyia* Ren, 1998, syn. n.), *Sinallomyia* nom. n. (pro n *Allomyia* Ren, 1998).

##### Diagnosis.

R1 rather long (four-fifths or more of wing length). R2+3 relatively straight. Crossvein r-m meeting R4+5 distad to Rs fork. Female cerci segmented, not foliaceous.

#### Subfamily Uranorhagioninae

KY Zhang, Yang & Ren, 2010 stat. n.

Uranorhagionidae KY Zhang, Yang et Ren, 2010, p. 564
Mostovskisarginae JF Zhang, 2010, p. 310, syn. n.

##### Type genus.

*Uranorhagio* KY Zhang, Yang et Ren, 2010

##### Included genera.

*Daohugosargus* gen. n. and *Uranorhagio* KY Zhang, Yang et Ren, 2010 (=*Mostovskisargus* JF Zhang, 2010; *Strenorhagio* KY Zhang, Yang et Ren, 2010).

##### Diagnosis.

R2+3 significantly bent. Position of r-m inconstant, distad to, just at, or basad to, Rs fork. Female cerci foliaceous, unsegmented.

#### Genus Uranorhagio

KY Zhang, Yang & Ren, 2010

http://species-id.net/wiki/Uranorhagio

Uranorhagio KY Zhang, Yang et Ren, 2010, pp. 564, 565
Strenorhagio KY Zhang, Yang et Ren, 2010, p. 566, syn. n.
Mostovskisargus JF Zhang, 2010, p. 310, syn. n.

##### Type species.

*Uranorhagio daohugouensis* KY Zhang, Yang et Ren, 2010

##### Included species.

*Uranorhagio asymmetricus* (KY Zhang, Yang et Ren, 2010) comb. n. (=*Strenorhagio conjugovenius* KY Zhang, Yang et Ren, 2010, syn. n.; *Mostovskisargus portentosus* JF Zhang, 2010, syn. n.; *Mostovskisargus signatus* JF Zhang, 2010, syn. n.), *Uranorhagio deviatus* (KY Zhang, Yang et Ren, 2010) comb. n. (=*Strenorhagio grimaldi* KY Zhang, Yang et Ren, 2010, syn. n.), besides the type species.

##### Redefinition.

R1 relatively short (some four-fifths of wing length). R2+3 strongly arched basally. Origin of Rs basad to d base. Rs fork basad to midlength of d. Crossvein r-m inconstant: slightly distad to, or just at, or somewhat basad to, Rs fork. M with four terminal branches. M1+2 furcated distad to d end.

#### Genus Daohugosargus gen. n.

urn:lsid:zoobank.org:act:2A134429-08CE-4D3F-8CBA-7ACD933A8620

http://species-id.net/wiki/Daohugosargus

##### Type species.

*Sharasargus eximius* KY Zhang, Yang & Ren, 2008

##### Included species.

The type species only.

##### Derivation of name. 

Chinese, *Daohugou*, alluding to the fossil locality, and *sargus*, a common ending in archisargid genera (the masculine gender).

##### Diagnosis.

R1 long (more than four-fifths of wing length). R2+3 short, significantly S-shaped. Origin of Rs distad to d base. Rs fork nearly at level of d end. Crossvein r-m far basad to Rs fork, and meeting anterior margin of d distad to its midlength. M with three terminal branches. M1+2 bifurcated basad to d end. Cell cu open.

##### Remarks.

The present new genus differs from *Uranorhagio* by the short, S-shaped R2+3; the origin of Rs, which is distally of d base, the position of r-m, which is far basad to Rs fork, and meeting anterior margin of d distad to its midlength; and by the M with three terminal branches, on which M1+2 fork basad to d end.

On the other hand, *Daohugosargus* gen. n. is distinct from *Sharasargus* Mostovski, 1996 in that: origin of Rs distad to d base; very short, S-shaped R2+3; first fork of Rs nearly at level of d end; and r-m meeting Rs stem far basad to Rs fork.

## Discussion

KY Zhang et al. (2010a) erected a new family, Uranorhagionidae KY Zhang, Yang et Ren, 2010 including five new species referred to two new genera based on several specimens from the “Daohugou Formation” in the vicinity of Daohugou, Inner Mongolia of China (not the true Jiulongshan Formation). They assigned Uranorhagionidae to the superfamily Tabanoidea, and considered Uranorhagionidae exhibiting a mixture of distinct characteristics of two families, the Rhagionemestriidae (Nemestrinoidea) and the Rhagionidae (Tabanoidea), but failed to discuss the relationship of Uranorhagionidae and Archisargidae (Archisargoidea).

**Figure 5.**
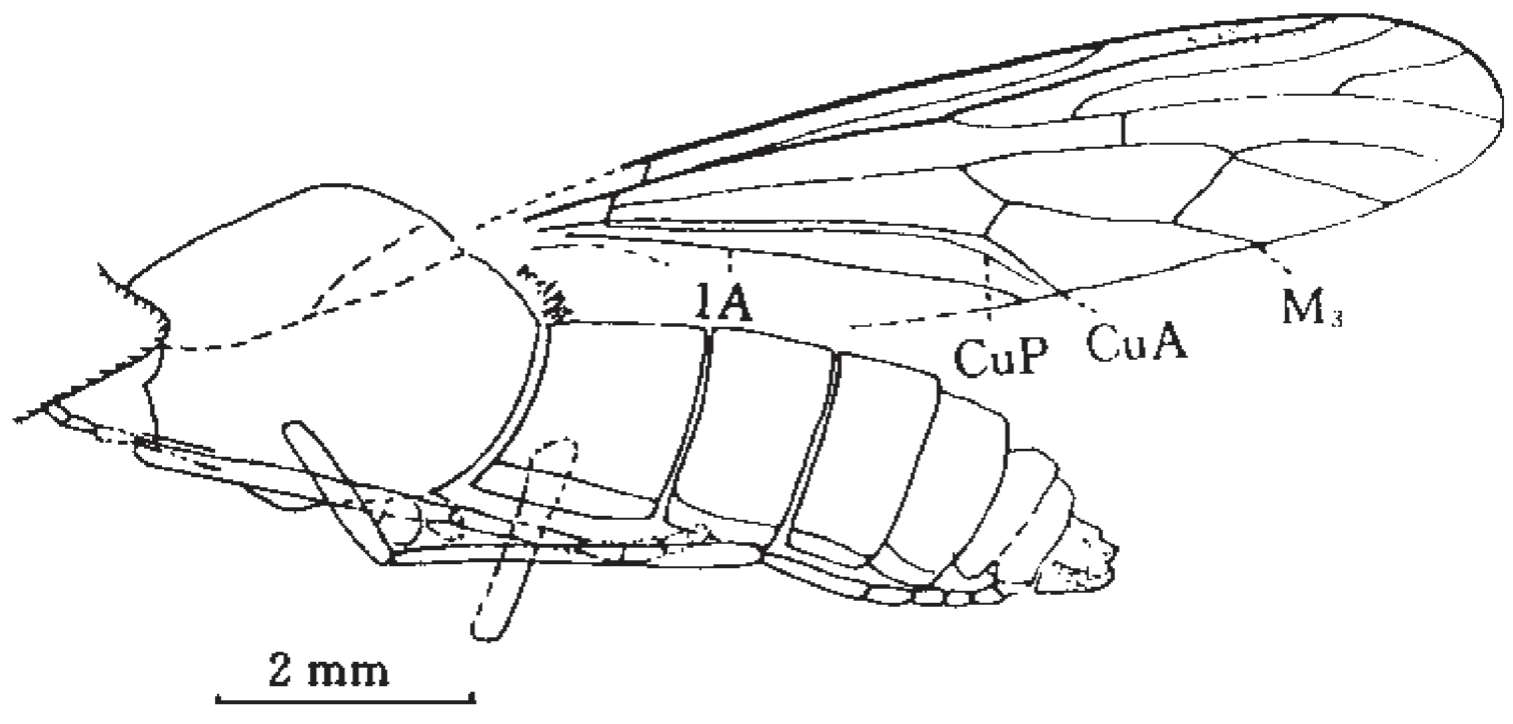
*Sharasargus oresbius* (Ren, 1998) comb. n. (originally *Pauromyia oresbia* Ren, 1998; after Ren 1998).

Comparing Uranorhagionidae with the Archisargidae, however, almost all the characteristics derived from body structures and wing venation in the former family are very closely similar to the latter family. The major difference of significantly bent R2+3 also demonstrates close resemblance to that of *Daohugosargus eximius*, an undoubted representative of archisargids although its systematic position at generic level is debatable (see Figure 4). It should be noted that the position of r-m in Uranorhagionidae is unstable: in some species slightly distad to, or just at, in other species slightly basad to, Rs fork. Nevertheless, this character also exists in Archisargidae (most representatives versus *Daohugosargus eximius*). As far as the petiolate M1+2 behind d end is concerned, there are some members of archisargids with M1+2 fork just at d end [see Figure 5, *Sharasargus oresbius* (Ren, 1998), comb. n., originally *Pauromyia oresbia* Ren, 1998], which does belong rather to Archisargidae than to Rhagionidae (detailed discussion, see below); and is more or less similar to that of uranorhagionids. The distal position of the M1+2 fork with respect to the d cell distal end may be a unique feature of uranorhagionids, which has the taxonomic significance only at generic, at most subfamilial, rank. Thus, Uranorhagionidae is a junior synonym for Archisargidae, and could be degraded as a subfamily referred to Archisargidae.

KY Zhang et al. (2010a) described five new species respectively assigned to two new genera: *Uranorhagio daohugouensis*, *Strenorhagio deviatus*, *Strenorhagio grimaldi*, *Strenorhagio asymmetricus* and *Strenorhagio conjugovenius*. However, these impressions demonstrate close similarities in body structures and wing venation each other, and then could be united into three species referred to a single genus: *Uranorhagio daohugouensis*, *Uranorhagio deviatus* (KY Zhang et al., 2010) and *Uranorhagio asymmetricus* (KY Zhang et al., 2010). The genus *Strenorhagio* KY Zhang et al., 2010 could be synonymized with *Uranorhagio* KY Zhang et al., 2010. The *Strenorhagio grimaldi* and *Strenorhagio conjugovenius* are synonyms for *Uranorhagio deviatus* and *Uranorhagio asymmetricus*, respectively. It should be noted that minor differences in the wing venation should be attributed to individual variation, which can be quite substantial in the extinct archisargids. For example, the holotype of *Uranorhagio asymmetricus* (CNU-DIB-NN2007020) (KY Zhang et al. 2010, p. 569, fig. 10) shows the r-m clearly basad to Rs fork in its left wing, but just at Rs fork in its right wing. That is to say the position of r-m being inconstant not only in different individuals but even in a single specimen.

The author (JF Zhang, 2010a) described two new species referred to a new genus within a new subfamily based on two specimens from the “Daohugou Formation” in the vicinity of Daohugou, Inner Mongolia of China, and assigned them to Archisargidae — Mostovskisarginae: *Mostovskisargus*: *Mostovskisargus portentosus* and *Mostovskisargus signatus*. However, these taxa were published somewhat later (publication date: 16 March, 2010) than those described by KY Zhang et al. (26 February, 2010). Thus, Mostovskisarginae, *Mostovskisargus*, *Mostovskisargus portentosus* and *Mostovskisargus signatus* are junior synonyms of Uranorhagioninae, *Uranorhagio* and *Uranorhagio asymmetricus*, respectively.

**Figure 6.**
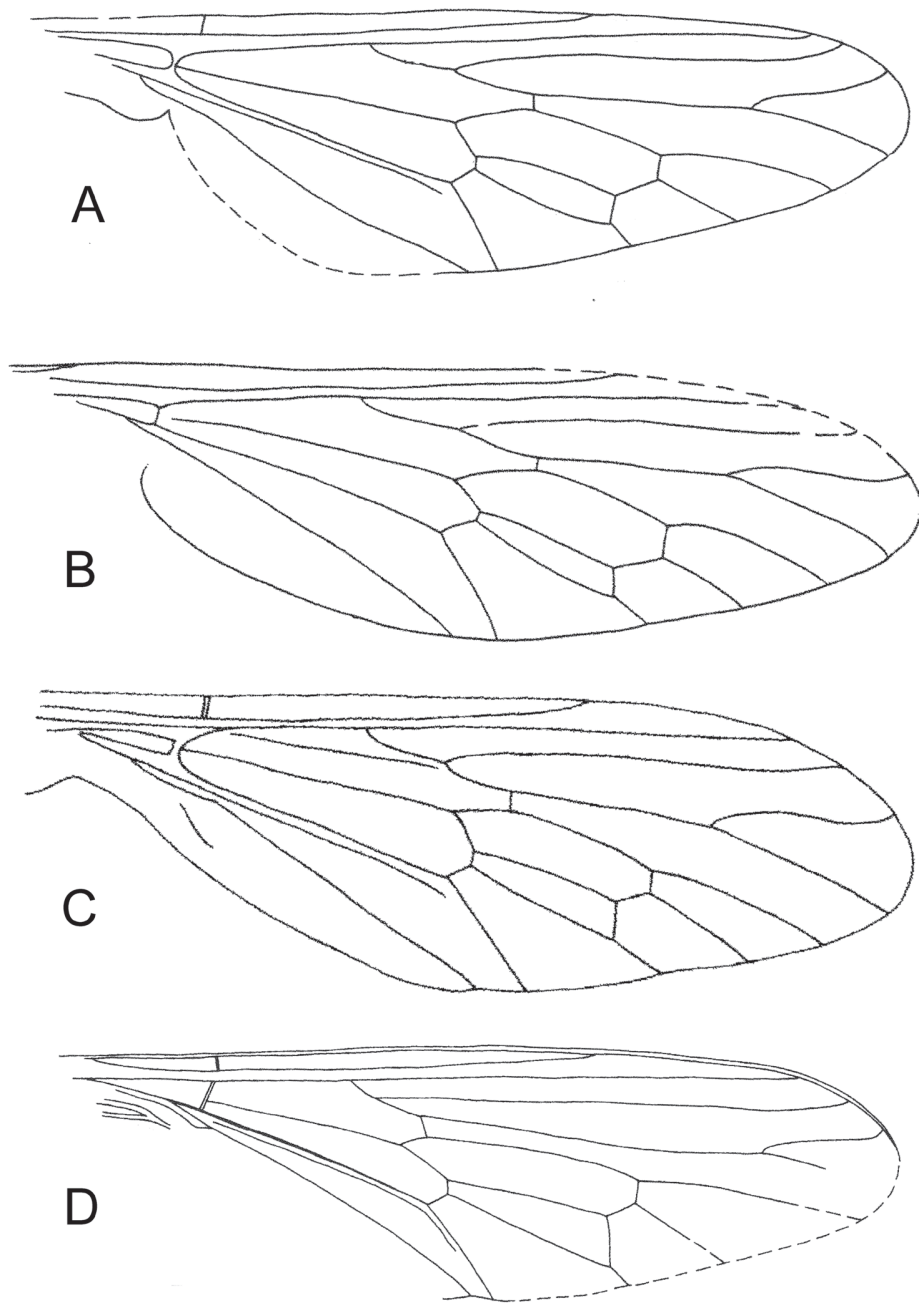
Similarity between four set of wings **A** *Mesosolva sinensis* KY Zhang et al., 2010 (after KY Zhang et al., 2010) **B** *Mesosolva sinensis* KY Zhang et al., 2010 (originally *Mesosolva jurassica* KY Zhang et al., 2010; after KY Zhang et al., 2010) **C** *Mesosolva sinensis* KY Zhang et al., 2010 (originally *Mesosolva jurassica* KY Zhang et al., 2010; after KY Zhang et al., 2010) **D** *Mesosolva zhangae* (KY Zhang et al., 2010) nom. n. (originally *Brevisolva daohugouensis* KY Zhang et al., 2010; after KY Zhang et al., 2010).

**Figure 7.**
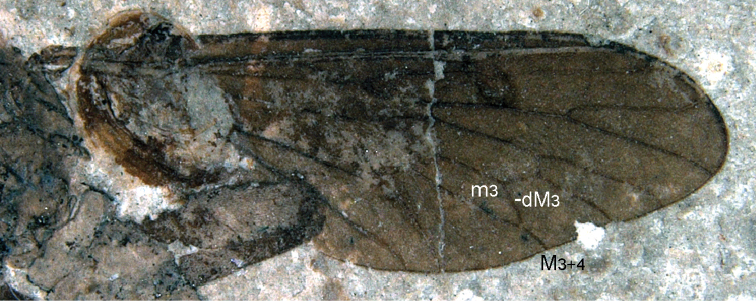
An undescribed wing of *Mesosolva* NIGP DHG902.

**Figure 8.**
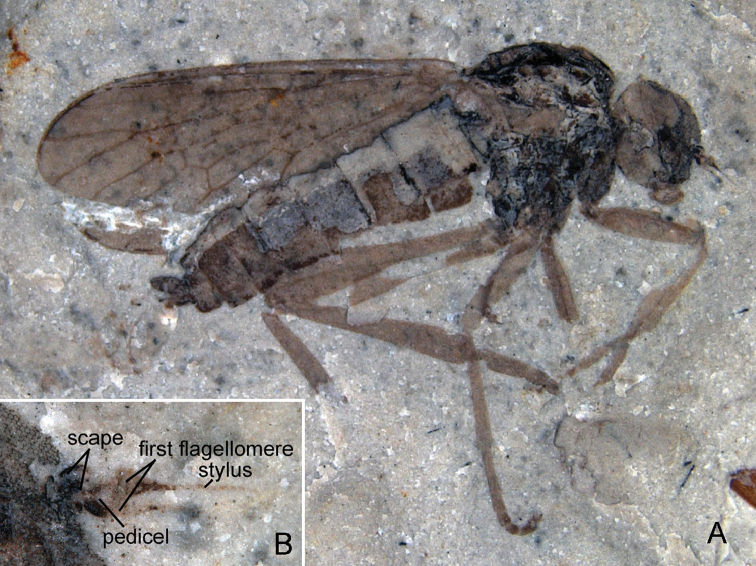
An undescribed male impression of *Mesosolva* NIGP DHG903, **A** habitus (lateral view) **B** antennae.

KY Zhang et al. (2010b) described a new species referred a new genus *Brevisolva daohugouensis* and two additional *Mesosolva* species: *Mesosolva jurassica* and *Mesosolva sinensis*. Judging from the original descriptions, drawings and photographs (KY Zhang et al., 2010b, pp.76–79, figs 1–8), *Mesosolva jurassica* demonstrates very close resemblance in body structures and wing venation to *Mesosolva sinensis*, and then, both species should be united into a single one (see Figure 6A–C herein). It should be repeatedly emphasized: due to individual variation and/or sexual dimorphism, some minor differences in the wing venation usually occur within an archisargid species. Such differences may exist even between the left and right wings of a single specimen. It is evident that *Mesosolva sinensis* is closely similar in body structure and wing venation to *Mesosolva daohugouensis* JF Zhang et HC Zhang, 2003. Both species are from the same fossil site. It might be debatable whether the two species could also be united into one species. On account of the characteristic wing venationof *Brevisolva daohugouensis* (see Figure 6D) showing close similarities to *Mesosolva sinensis* it is difficult to see how the genus *Brevisolva* could be separated. The genus *Brevisolva* as defined by its authors (KY Zhang et al, 2010b) does not have diagnostic features that separate it from *Mesosolva*. As for the short Rs stem, short R5, the position of r-m which is close to d base, these characteristics in wing venation of *Brevisolva* could be treated as the difference between species, and are also similar respectively to some known species of *Mesosolva*, for example, in *Mesosolva longivena* Mostovski, 1996 and *Mesosolva balyshevae* Mostovski, 1996. Additionally, the short petiole of cell m3 is not the particular feature of *Brevisolva*. There is an undescribed impression of *Mesosolva* showing its petiole clearly shorter than the section dM3 (see Figure 7). *Brevisolva daohugouensis* could be regarded as a species of *Mesosolva*. A new specific name, *Mesosolva zhangae* (KY Zhang et al., 2010), nom. n., is proposed because the *Mesosolva daohugouensis* has already been occupied (JF Zhang and HC Zhang 2003).

Hong (1983) described two monobasic genera, *Mesosolva* and *Prosolva* from the Callovian–Oxfordian Haifanggou Formation in Beipiao, Liaoning, China. He assigned these to the family Xylomyiidae (originally Solvidae). JF Zhang et al. (1993) discussed the systematic position of *Mesosolva parva* Hong, 1983 and *Prosolva huabeiensis* Hong, 1983, pointed out that these probably belong in an unnamed group at familial level, which is probably related to the family Rhagionidae based on the characteristics of antenna; but the structures of antenna were mistakenly described: the so-called second segment (i.e. pedicel) is, in fact, the third segment (i.e. first flagellomere) (JF Zhang et al., 1993, p. 667). In the author’s collection of brachycerous flies from Daohugou biota, there is another nearly complete impression of male *Mesosolva* with antennae visible, which consist of the scape, pedicel, first flagellomere, and stylus (Figure 8). It is evident that the so-called pedicel described by Hong (1983) is the first flagellomere, in size and shape very closely resembling that of the present undescribed specimen (see Figure 8), although they are from different individuals at different fossil localities. Mostovski (1996a, 1996b) redefined Archisargidae and *Mesosolva*. Meanwhile, he transferred *Mesosolva* into Archisargidae, and described seven new *Mesosolva* species. He thought that *Mesosolva parva* and *Prosolva huabeiensis* probably belong to the same genus (Mostovski 1996b). JF Zhang and HC Zhang (2003) described the first record of *Mesosolva* from the Daohugou biota, and agreed with Mostovski’s (1996b) conclusion mentioned above. Recently, KY Zhang et al. (2010b) revised the diagnosis of *Mesosolva* proposed by Mostovski (1996b). Unfortunately, the redefinition is unsatisfactory. What is striking is the additional characteristic: CuA1 arising from cell bm, mouth of cell sc wide open, much wider than that of cells r1 and r2+3. They failed to explain how these features could be defined as the *Mesosolva* diagnosis. Actually, these that they added are common characteristics of archisargid genera, and occur in almost all representatives referred to various genera in the two subfamilies (Archisarginae and Uranorhagioninae) of Archisargidae. The author argues that these delineations proposed by KY Zhang et al. (2010) do not conform to the diagnoses of all the archisargid genera.

**Figure 9.**
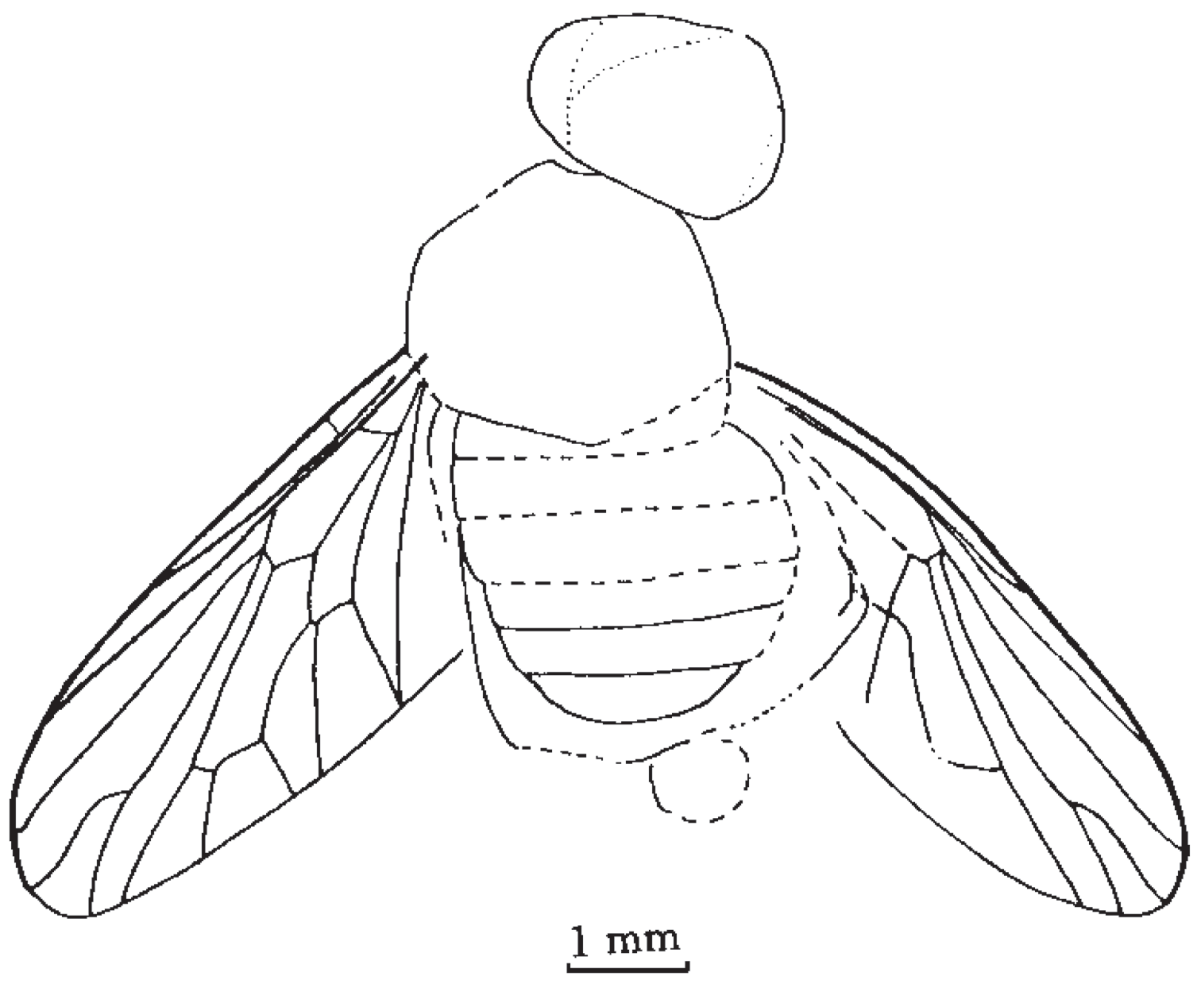
*Sinallomyia ruderalis* (Ren, 1998) (originally *Allomyia ruderalis* Ren, 1998; after Ren 1998).

*Sinallomyia ruderalis* from the Lower Cretaceous Yixian Formation was originally regarded as a new genus and species of the subfamily Tabaninae within Tabanidae. Judging from the original illustration (Ren 1998, p. 69, fig. 6; Figure 9 herein) the R1 is some four-fifths of wing length; and the venation and body structure demonstrate, more or less, resemblance to *Mesosolva zhangae* (KY Zhang et al., 2010), nom. n.; hence, this genus and species can be transferred to the Archisarginae of Archisargidae (JF Zhang, 2012a).

*Pauromyia oresbia* from the same locality and horizon was previously assigned to the Rhagionidae (Ren 1998, p. 72, fig. 11; Figure 5 herein). This species can be moved into *Sharasargus* within Archisarginae, Archisargidae because its venation shares close similarity to *Sharasargus spiniger* Mostovski, 1996 referred to Archisarginae, Archisargidae (JF Zhang, in press).

**Figure 10.**
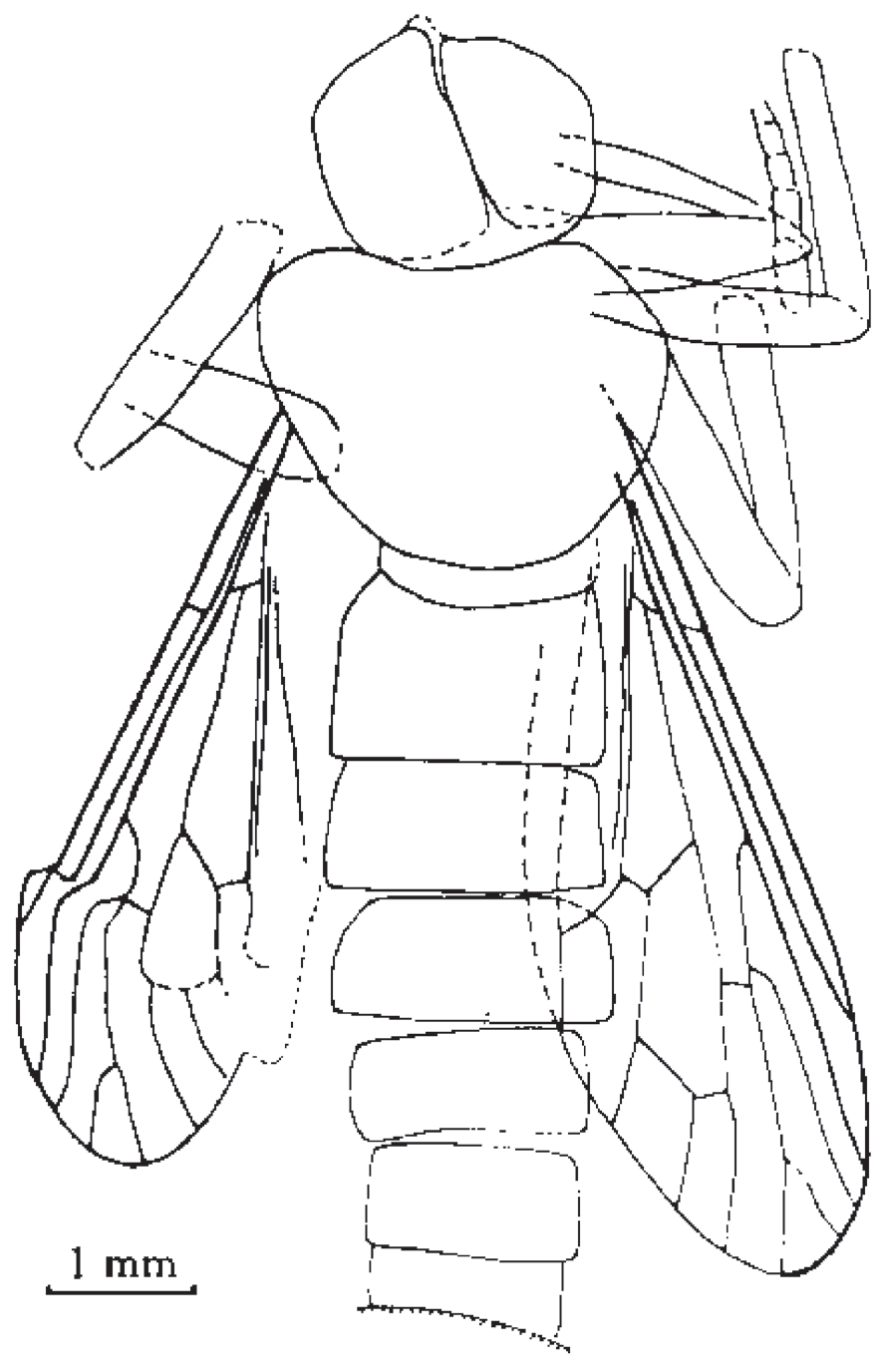
*Ovisargus (Helempis) yixianensis* (Ren, 1998) comb. n. (originally *Helempis yixianensis* Ren, 1998; after Ren 1998).

**Figure 11.**
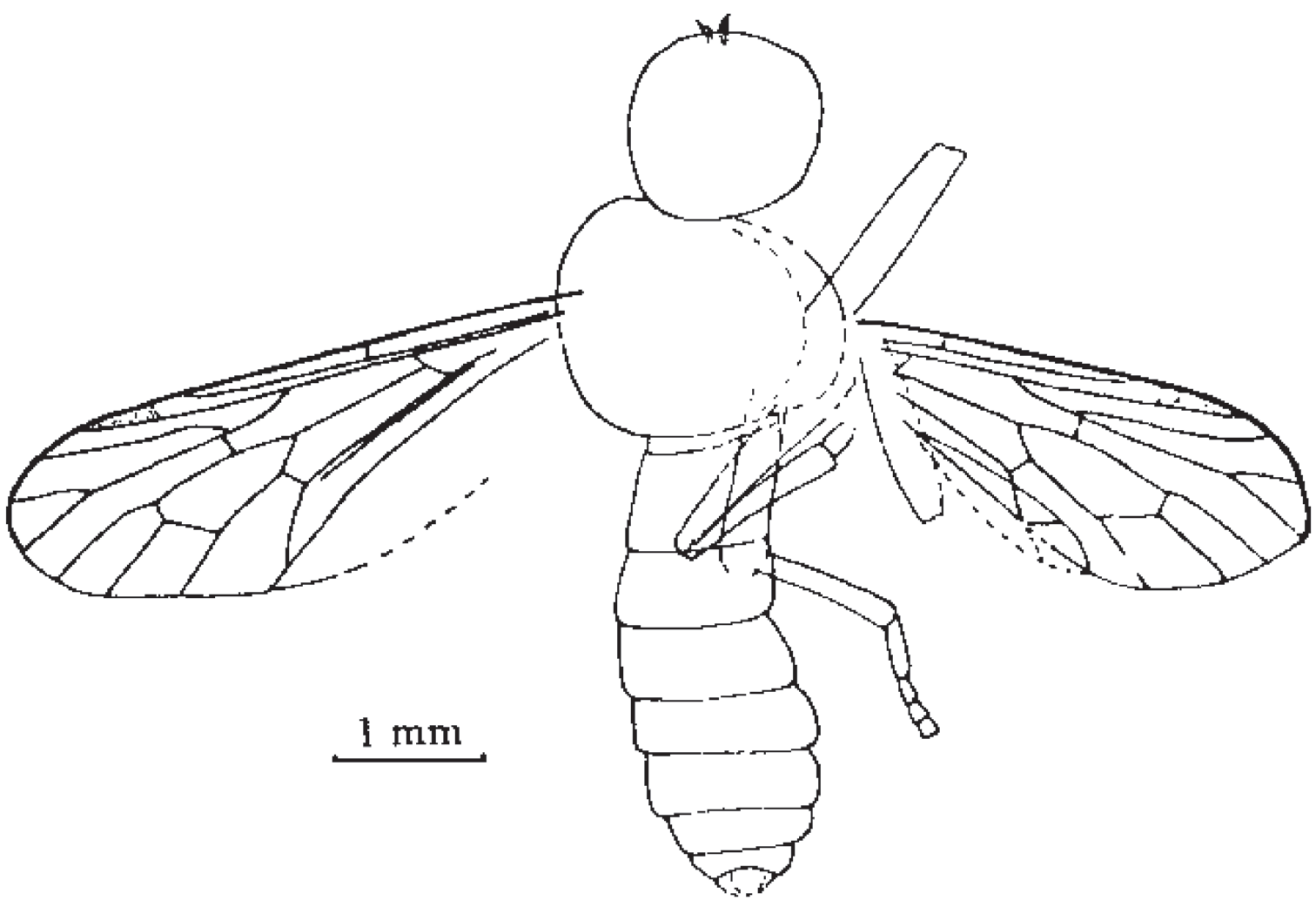
*Ovisargus (Helempis) yixianensis* (Ren, 1998) comb. n. (originally *Helempis eucalla* Ren, 1998; after Ren 1998).

Ren (1998) described two new species of a new genus: *Helempis yixianensis* and *Helempis eucalla* from the same locality and horizon. He considered these taxa having typical wing venation of Protempididae. On the basis of original drawings (Ren 1998, pp. 80, 81, figs. 22, 23; Figures 10, 11 herein), the two species which might be probably united into one species have very long R1, which is some four-fifths (or more) of wing length, relatively narrow and long wings, unsegmented arista, and the characteristic discoidal cell, which is distinctly shifted distally. All the characters contradict to including these species in the Protempididae. On the contrary, *Helempis yixianensis* and *Helempis eucalla* demonstrate close resemblance in wing venation to *Ovisargus gracilis* Mostovski, 1996, and then could be transferred to *Ovisargus* Mostovski, 1996 (Archisarginae, Archisargidae). A detailed discussion will be made in a separate paper.

The *Origoasilus* KY Zhang et al., 2011 previously erected as a new genus and assigned to a new family Origoasilidae KY Zhang et al., 2011 has been transferred to Archisarginae of Archisargidae. The Origoasilidae is a junior synonym for Archisargidae (JF Zhang, 2012b).

Ideally, these previously described species from the upmost Middle–lowest Upper Jurassic “Daohugou Formation” and the Lower Cretaceous Yixian Formation should be revised properly through re-examination of the type material, since the original drawings may contain details resulted from misinterpretation of insufficiently preserved structures. For this reason, until such time as reinvestigation of these specimens is possible, their taxonomic positions could be temporarily assigned to Archisarginae, Archisargidae based on original descriptions and drawings because the Mesozoic archisargid flies have characteristic wing venation (see revised diagnosis of Archisargidae mentioned above) which is easily separated from other extinct and extant families within the lower Orthorrhapha, Brachycera.

## Supplementary Material

XML Treatment for Orientisargidae

XML Treatment for Orientisargus

XML Treatment for Orientisargus illecebrosus

XML Treatment for Archisargidae

XML Treatment for Archisarginae

XML Treatment for Uranorhagioninae

XML Treatment for Uranorhagio

XML Treatment for Daohugosargus
